# Mental health of children and young people aged 5-16 in England: socio-demographic and clinical characteristics associated with support and service contact

**DOI:** 10.1192/j.eurpsy.2023.1216

**Published:** 2023-07-19

**Authors:** S. P. Trethewey, F. Mathews, A. Russell, T. Newlove-Delgado

**Affiliations:** Children and Young People’s Mental Health (ChYMe) research collaboration, University of Exeter, Exeter, United Kingdom

## Abstract

**Introduction:**

Mental health problems are common in children and young people (CYP) in England, yet evidence suggests high levels of unmet need in this group. Understanding of the determinants of mental health-related service contact is needed to identify gaps in service provision and areas for targeted intervention to improve access.

**Objectives:**

To determine the relationship between CYP characteristics and mental health-related support and service contact in England.

**Methods:**

A secondary analysis of the 2017 NHS Digital Mental Health of Children and Young People (MHCYP-2017) cross-sectional survey dataset was performed. MHCYP-2017 was a national survey investigating the mental health of CYP using a stratified multistage random probability sampling approach, providing the official national statistics for England. Multi-informant data were collected through a combination of questionnaires and interviews. Expert clinical rating took place to formally identify the presence of mental disorders, according to established diagnostic criteria. This secondary analysis describes mental health-related support and service contact amongst 6681 participants aged 5-16 recruited to the MHCYP-2017 study. A range of socio-demographic and clinical characteristics were analysed as explanatory variables and their relationships with different types of support/service contact were examined through multivariable multinomial logistic regression. Analyses were stratified by age group: 5-10- and 11-16-year-olds.

**Results:**

Overall, around 25% of parents reported CYP mental health-related contact with one or more types of support/service in the past 12 months due to concerns regarding CYP “emotions, behaviour, concentration or difficulties in getting along with people”. Age stratified multivariable analyses revealed several statistically significant associations between participant socio-demographic/clinical characteristics and mental health-related support and service contact, independent of CYP mental health status and parental perception of difficulties. These associations were not necessarily consistent across mental health support categories, suggesting that several of the measured characteristics have differential relationships with different types of support and service contact. Whilst there were some differences between the 5-10 and 11-16 age groups, similar associations were seen for many of the explanatory variables. Socioeconomically disadvantaged and black and minority ethnic CYP were less likely to have had professional contact for mental health problems.

**Conclusions:**

There may be higher levels of unmet need in socioeconomically disadvantaged and black and minority ethnic CYP, warranting further investigation and efforts to address inequalities. Further longitudinal studies are needed to elucidate causal associations and mechanisms underlying these observations.

**Disclosure of Interest:**

None Declared

